# T Lymphocytes, Multi-Omic Interactions and Bronchopulmonary Dysplasia

**DOI:** 10.3389/fped.2021.694034

**Published:** 2021-06-08

**Authors:** Gergely Toldi, Helmut Hummler, Thillagavathie Pillay

**Affiliations:** ^1^Institute of Immunology and Immunotherapy, University of Birmingham, Birmingham, United Kingdom; ^2^Liggins Institute, University of Auckland, Auckland, New Zealand; ^3^Department of Neonatology, University of Tuebingen, Tuebingen, Germany; ^4^Faculty of Science and Engineering, University of Wolverhampton, Wolverhampton, United Kingdom; ^5^Department of Neonatology, University Hospitals Leicester NHS Foundation Trust, Leicester, United Kingdom; ^6^College of Life Sciences, University of Leicester, Leicester, United Kingdom

**Keywords:** antibiotics, corticosteorids, gut-lung axis, hyperoxia, inflammation, microbiome

## Abstract

Bronchopulmonary dysplasia (BPD) remains a significant clinical challenge in neonatal medicine. BPD is clearly a multifactorial disease with numerous antenatal and postnatal components influencing lung development. Extremely immature infants are born in the late canalicular or early saccular stage and usually receive intensive care until the early alveolar stage of lung development, resulting in varying magnitudes of impairment of alveolar septation, lung fibrosis, and abnormal vascular development. The interactions between T lymphocytes, the genome and the epigenome, the microbiome and the metabolome, as well as nutrition and therapeutic interventions such as the exposure to oxygen, volutrauma, antibiotics, corticosteroids, caffeine and omeprazole, play an important role in pathogenesis and disease progression. While our general understanding of these interactions thanks to basic research is improving, this knowledge is yet to be translated into comprehensive prevention and clinical management strategies for the benefit of preterm infants developing BPD and later during infancy and childhood suffering from the disease itself and its sequelae. In this review, we summarise existing evidence on the interplay between T lymphocytes, lung multi-omics and currently used therapeutic interventions in BPD, and highlight avenues for potential future immunology related research in the field.

## Introduction

The progressive, preferential maturation of the neonatal adaptive immune system is driven by multi-omic interactions *in-utero* and after birth. Together, these shape health and disease in the newborn and infant. After birth, the neonatal adaptive immune system needs to fulfil the requirements of two distinct developmental needs. On one hand, it needs to recognise and eliminate pathogens to prevent infections. On the other hand, it needs to establish tolerance towards harmless antigens and bacteria that will form the microbiome at various mucous membranes of the body (1). This delicate developmental program may be influenced by several factors, such as the external environment, prematurity, and measures provided to ensure survival. These factors can easily shift the fine-tuned balance that aims to maintain the progression of both immunological developmental needs at the same time. While our understanding of such interactions in the context of the gut microbiome and necrotising enterocolitis is improving (2), there is still a substantial knowledge gap around the role of the immune—microbiome, and multi-omic interactions in contributing to the development of other pathologies, such as bronchopulmonary dysplasia (BPD). This complex lung condition is becoming increasingly prevalent especially among extremely preterm neonates due to the decreasing gestational age thresholds for survival (3).

A recent comprehensive review by Thebaud et al. summarised the essence of the pathophysiology of BDP as the “impaired postnatal adaptation of the preterm lungs to breathing challenged by initial lung injury due to surfactant deficiency, exposure to hyperoxia, mechanical ventilation, inadequate nutrition, infection and inflammation, which together provide considerable hurdles to normal pulmonary growth and repair and result in a loss of effective alveolar surface area” (4). Lal et al. have lately suggested that BPD is unlikely to be a single entity, or even a spectrum of disease with the same pathophysiologic background. The common denominator is the affected population of premature infants in the late canalicular, saccular or early alveolar stage of lung development, resulting in varying degree of alveolar septation impairment, lung fibrosis, and abnormal vascular development (5). Instead of a single chain of events, an interplay between the immune system, the genome, epigenome, microbiome, proteome, metabolome, and environmental factors, such as oxygen, mechanical ventilation and medication including antibiotic therapy, may contribute to the pathogenesis to different degrees. The pathophysiology of BPD may be deeply intertwined with these multi-omic interactions. The potential importance of immune-mediated mechanisms guiding these interactions leading to and responsible for recovery from lung damage are poorly understood and necessitate future immunological research in this area.

The diverse population of T lymphocytes is the cornerstone of adaptive immunity. They play a key role not only in recognising and eliminating pathogens, but also in coordinating tissue inflammatory responses and repair. The functional heterogeneity of T cells allows for a fine-tuned control system engaging both pro- and anti-inflammatory mechanisms mediated by relevant T lymphocyte subsets.

In this review, we summarise existing evidence on the interplay between T lymphocytes, lung multi-omics, currently used therapeutic interventions and BPD. We aim to visualise this disease through the lens of an immunological approach and highlight avenues for potential future immunology related research in BPD.

## T Lymphocytes

Limited studies in human neonates show some alteration in T lymphocyte numbers, activation and regulation in preterm babies who develop BPD. Ballabh et al. compared the proportion of lymphocyte subsets using flow cytometry in preterm infants with RDS who later developed BPD in comparison with those who did not. The absolute number of lymphocytes and CD4 cells was lower among infants with BPD over the first 2 weeks of life The proportion of CD8 and NK cells was not different between the two groups. The proportion of B cells was decreased in BPD infants only on day 7. The proportion of CD4 cells expressing CD62L was selectively reduced in BPD infants, indicating L-selectin shedding, a marker of T cell activation (6). In a similar study, Turunen et al. reported that in preterm infants with RDS, the increased proportion of activated T cells predicted the development of BPD. RDS infants who subsequently developed BPD had higher CD54 activation marker expression on CD4 cells on day 3 of life and on CD8 cells on days 1 and 3 of life as compared with infants without BPD (7).

Jackson et al. analysed blood samples drawn from preterm infants born at 28 weeks of gestation or less at the first, second, and fourth week of life. Leukocytes isolated from infants who developed BPD had increased PMA/ionomycin stimulated and unstimulated IL-4 mRNA expression at the first week of life, while that of IFN-g was comparable. The expression of IL-2 was also decreased in infants developing BPD, however, this difference was more sustained. The Th17 and regulatory T cell immune profile was comparable in infants who developed BPD and in those who did not (8). On the contrary, in cord blood samples of infants born at 32 weeks of gestation or less, Misra et al. identified a decrease in CD4 cells and CD4+ FoxP3+ CD25+ CD127lo regulatory T cells using flow cytometry in those who later developed BPD (9).

Using a baboon model, Rosen et al. investigated whether autoreactive T cells could contribute to lung injury in BPD. In their functional studies, they performed mixed lymphocyte reactions using thymocyte or splenocyte responders with autologous lung cells as the stimulators. BPD thymocytes and splenocytes showed an increased proliferative response to autologous lung cells compared to controls. 2A11, an anti-bombesin antibody known to protect against BPD in baboons, normalised this response and the generation of autoreactive T cells that could contribute to lung injury (10).

The abundance of IL-1 strongly correlates with the long-term outcome and severity of BPD. Nold et al. demonstrated that the anti-inflammatory IL-1 receptor antagonist (IL-1Ra) prevented BPD precipitated by lipopolysaccharide and hyperoxia in a murine model. IL-1Ra treatment for 28 days inhibited the evolvement of histological changes characteristic for BPD (11). Furthermore, it also prevented the increase in pulmonary vascular resistance and pulmonary dysangiogenesis. Importantly, this protection against pulmonary hypertension was maintained at day 60 of life in mice (12).

The human lung harbours CD4+ and CD8+ tissue resident memory T cells (TRM) that persist in stable frequencies for decades throughout the individual's lifespan. Studies in adults revealed their important contribution to pathogen defence, tissue homeostasis and immune surveillance. They also play a role in promoting inflammation in asthma (13). During infancy, naive T cells represent the predominant population not only in peripheral blood but also in the lungs (14). With repeated pathogen and harmless environmental antigen exposures through inhalation and aspiration, effector memory T cells outnumber naïve T cells in the lung by late childhood, along with coincident expression of TRM phenotypes (15). In spite of their important role in lung pathology, probably due to difficulties of tissue sample collection from preterm patients, their contribution to the pathogenesis of BPD has not been elucidated.

Most of the alterations observed in lymphocyte subsets and cellular or humoral biomarkers have been shown to have poor predictive accuracy, and do not considerably improve the predictive ability of clinical variables, such as gestational age, birth weight or respiratory parameters (5) in relation to the development of BPD. On the other hand, most of these observational studies were not designed or powered to assess the predictive value of these markers. The application of the latest multi-omic approaches might provide added insights towards understanding the immune mechanisms by which BPD develop. In doing so, it could contribute towards fine tuning immune based predictive markers for the development, prevention and limitation of the severity of BPD in premature neonates.

## Multi-Omic Interactions

### Genome and Epigenome

Pietrzyk et al. compared whole genome expression using microarrays in blood samples of preterm infants with and without BDP on days 5, 14 and 28 of life. Two thousand eighty six genes were differentially expressed on day 5, while 324 on day 14 and 3,498 on day 28, respectively. Pathway enrichment analysis revealed that the cell cycle pathway was up-regulated in BPD. Four pathways related to inflammatory response (T cell receptor signalling, primary immunodeficiency, hematopoietic cell lineage, and cell adhesion molecules) were down-regulated in BPD at all three time points. The expression of these genes correlated with both immaturity and disease severity. The most significantly down-regulated pathway was the T cell receptor signalling pathway (16).

Revhaug et al. examined the regulatory relation between immune gene expression and DNA methylation after hyperoxia for 14 days followed by a recovery period of 14 days in air using a neonatal mouse model of BPD. A high proportion of immune genes showed significant alterations in expression. For instance, B cell genes involved in the cytokine-cytokine receptor interaction, the PI3K-AKT, and the B cell receptor signalling pathways were found to be altered. They also observed significant DNA hypermethylation in the PI3K-AKT pathway and other immune genes. Some of these could also influence the differentiation and function of the T lymphocyte subset. The genomic and epigenomic influence of oxygen exposure could therefore play a role in the pathogenesis of BPD and the increased susceptibility to respiratory infections seen in premature babies with BPD (17).

Further work is needed to explore consistent patterns of the contribution genomic and epigenomic changes of T lymphocytes and other immune cells to the pathogenesis of BPD.

### Microbiome and Metabolome

The airway microbiome evolves over time with increasing bacterial loads and diversity. Staphylococcus, Ureaplasma and, according to some reports, Acinetobacter are the predominant genera early after birth. BPD is associated with increased microbial community turnover. Alterations in the relative abundance of Proteobacteria and Firmicutes, and decreased Lactobacilli were reported with the progression of BPD (18, 19).

Lal et al. reported an established diverse airway microbiome at birth in both preterm and term infants (20). Older infants with BPD had reduced airway microbial diversity (18, 20). The presence of members of the phylum Proteobacteria, such as *E. coli*, was associated with BPD pathogenesis. Furthermore, genus *Lactobacillus* was decreased at birth in infants with chorioamnionitis, an independent risk factor for BPD, and in preterm infants who subsequently developed BPD. Genus *Lactobacillus* is known to have anti-inflammatory properties in the airway (21) and was shown to regulate alveolar development in a mouse model (22).

The authors concluded that their results may suggest foetal acquisition of an airway microbiome which may prime the developing pulmonary immune system. Although based on earlier reports it was indeed presumed that the human placenta may harbour a microbiome, and, therefore the womb and the intrauterine environment may not be sterile, recent extensive work by de Goffau et al. elegantly demonstrated that the human placenta is in fact sterile and has no microbiome (23). Signals detected in non-culture based microbiological experiments were most likely related either to sampling issues, for instance, the acquisition of bacteria during labour and delivery, or to contamination of laboratory reagents with bacterial DNA. These limitations need to be cautiously taken into account when interpreting results from these reports and their implications on the potential antenatal development of airway microbiome in the foetus.

The lung microbiome was described to interact with the immature immune system by inducing the expression of CD274, a B7-family antigen on pulmonary dendritic cells, eliciting inhibition of T cell activation via CD279 (also known as PD-1 receptor) on T cells. Blocking pulmonary CD274 during the first 2 weeks of life induced a disproportionate inflammatory response to allergens later in life in mice (24).

The same group later reported plausible mechanisms by which the airway microbiome could modify the risk for BPD by identifying differentially abundant functional orthologue genes in infants predisposed to BPD and controls. They also identified metabolites that were differentially enriched in the two cohorts. Specific pathways were less abundant in the functional metagenome of the microbiota of BPD-predisposed infants compared to controls. Furthermore, the airway metabolome of BPD-predisposed infants was enriched for metabolites involved in fatty acid activation and androgen and oestrogen biosynthesis. These findings suggest that the early airway microbiome may alter the metabolome, thereby modifying the risk of BPD. They may also account for the previously described male gender predilection of BPD (25).

Another important mechanism *via* which the microbiome can influence immune cell function is through the production of microbial metabolites, including short chain fatty acids or tryptophane catabolites. Short chain fatty acids, in particular propionate and butyrate, are known to play an important role in promoting regulatory T cell differentiation and proliferation via the inhibition of histone deacetylases (26). Indoleamine 2,3-dioxygenase is responsible for the production of tryptophane catabolites which, amongst other functions, act as agonists for the aryl hydrocarbon receptor (AhR). AhR activation facilitates immune suppression through the production of IL-22 and also promotes the development of regulatory T-cells. A bacterial genus capable of metabolising tryptophane into AhR agonists are Lactobacilli (27).

Omeprazole was shown to achieve the same effect as tryptophane catabolites by inducing CYP1A1 possibly through an AhR-mediated process. Combined pre- and postnatal administration of omeprazole attenuated hyperoxic lung injury evoked in preterm rabbits (28). AhR signalling was found to be protective against hyperoxic injury in human foetal pulmonary microvascular cells and neonatal mice mediated by gene expression of immunomodulatory and developmental pathways (29).

Shivanna et al. tested whether omeprazole attenuated hyperoxia-provoked lung injury in adult mice by inducing CYP1A1 by activating pulmonary AhRs. Attenuation of lung injury by omeprazole paralleled enhanced pulmonary CYP1A1 in omeprazole treated mice. Omeprazole failed to enhance pulmonary CYP1A1 expression and protect against hyperoxic lung injury in AhR deficient mice (30). The same group went on to elucidate whether omeprazole protects against hyperoxia-induced lung injury in a neonatal mouse model of BPD by supposedly activating pulmonary AhRs. Surprisingly, in the neonatal period, hyperoxia-induced alveolar and pulmonary vascular simplification, inflammation, oxidative stress, and vascular injury were augmented rather than mitigated in omeprazole-treated animals. These findings were associated with decreased rather than increased pulmonary AhR activation (31).

Segal et al. revealed in their study that azithromycin increased levels of tryptophan catabolites in bronchoalveolar lavage fluid of smokers who developed emphysema, thus reducing the production of pro-inflammatory cytokines (32). Besides its potent anti-inflammatory properties, azithromycin is also known to be highly effective against Ureaplasma, a likely contributor to BPD pathogenesis. However, earlier trials on its use for the prevention of BPD were not adequately powered. In a randomised phase II trial of preterm infants between 24+0 and 28+6 weeks of gestation, a three-day course of azithromycin improved Ureaplasma-free survival and showed a promising trend towards a shorter exposure to invasive ventilation and supplemental oxygen and a shorter duration of hospitalisation (33). A currently ongoing RCT, the AZTEC study aims to investigate if a 10-day course of azithromycin improves rates of survival without CLD when compared with placebo (34).

There is increasing evidence on the role of the airway microbiome in BPD. However, these studies may have sampling and methodological limitations which should be observed when interpreting the results.

### Gut-Lung Axis

Epidemiological studies have linked perinatal antibiotic exposure in human newborns to an increased risk for BPD, possibly mediated through the gut-lung axis (35, 36). A recent important report on the gut-lung axis illuminated that intestinal commensal bacteria prevent pneumonia by promoting the trafficking of IL-22-producing group 3 innate lymphoid cells (ILC3) into the lung of newborn mice (37). The high level of IL-22 inhibits pathogen proliferation and regulates granulocyte colony-stimulating factor production in the lung (38). This trafficking is dependent on the presence of an intestinal microbiome and is mediated by CD103+CD11b+ intestinal dendritic cells that induce CCR4 homing receptor overexpression on intestinal IL-22+ ILC3. A chemokine expressed in the lung epithelium, CCL17, activates the CCR4 receptor. Thus, it promotes the trafficking of IL-22+ ILC3 into the lung. The authors confirmed these observations in a cohort of 15 human neonates treated with antibiotics and having a history of mechanical ventilation, finding reduced concentrations of IL-22 in their BAL fluid samples (37). The vast majority of IL-22 producers in both pups and human infants were ILC3s, in contrast with NK, Th17 and γδ T cells previously reported in adults (39–41).

Willis et al. have recently shown how perinatal maternal antibiotic exposure influenced lung injury in a hyperoxia-based mouse model of BPD. Antibiotic-induced intestinal dysbiosis during the perinatal period promoted a more severe BPD phenotype characterised by increased pulmonary fibrosis and overall mortality. This was associated with decreased IL-22 expression in bronchoalveolar lavage and was independent of hyperoxia-induced inflammasome activation. The results emphasise the transgenerational disruptive effects of antibiotic exposure on the inflammatory balance in the lung (42).

## Interventions

### Hyperoxia

While the provision of increased FiO2 is often necessary to ensure adequate tissue oxygenation in the preterm neonate, hyperoxia is known to have harmful pulmonary and extrapulmonary consequences and is associated with increased oxidative stress (43), morbidity, such as retinopathy of prematurity (44–46) and mortality (47). Oxygen levels influence both the developing microbiome and immune system, and we have gained increasing understanding of these interactions over the recent years. Anaerobic bacteria do not tolerate high levels of oxygen, thus hyperoxia can confer a selective growth advantage on aerobic bacterial species within the microbiome. Innate immune cells heavily rely on the generation of reactive oxygen and nitrogen species to elicit a response against pathogens and other stimuli. Therefore, inflammatory lung conditions are characterised by high levels of oxidative stress. The role of oxidative stress in the development of BPD is comprehensively reviewed by Capasso et al. (48).

In a mouse study, Kumar et al. demonstrated that neonatal hyperoxia suppresses the expression of genes involved in T and B cell activation, an effect still detectable in adulthood at 3 months. Newborn mice litters were exposed at 3 days to 85% O_2_ or room air for a period of 12 days. Whole lung mRNA was isolated at 2 weeks and 3 months. Further to the gene expression changes, adult mice exposed to neonatal hyperoxia also had lower levels of anti-inflammatory IL-4 and IL-10 in the lung at 3 months (49).

Ashley et al. investigated the effects of hyperoxia on the gut and lung microbiome in mice and human samples (50). In an observational study of critically ill patients receiving mechanical ventilation, as well as in experiments on neonatal and adult mouse models, they demonstrated that hyperoxia was associated with a selective growth advantage on oxygen-tolerant respiratory microbial species, such as *S. aureus*. During exposure of mice to hyperoxia, both the lung and gut microbiome was altered, and this preceded and contributed to oxygen-induced lung injury. Compared to normoxia, lungs of hyperoxia-exposed mice had decreased community richness and had increased relative abundance of an oxygen-tolerant Streptococcus species, and variations in lung and gut microbiota correlated with the severity of lung inflammation. Systemic treatment with broad Gram-negative spectrum antibiotics, ceftriaxone, selectively increased the severity of oxygen-induced lung injury as measured by alveolar protein excretion. Interestingly, germ-free mice were protected from oxygen-induced lung injury.

The authors highlighted that the specific contributions of lung and gut microbiota to the above effects are not fully understood. The same group demonstrated earlier that variation in lung innate immunity more strongly reflected lung than gut microbiota in mice (51). In the hyperoxia study, variation in lung inflammation correlated with the diversity and community composition of both lung and gut microbial communities, but correlation with lung microbial communities preceded correlation with gut microbial communities (24 vs. 48 h). Therefore, most likely both the gut and lung microbiome play important, temporally distinct roles in the pathogenesis of oxygen-induced lung injury.

Early airway dysbiosis and the disruption of the development of a normal pulmonary microbiome are associated with the development of BPD. In a recent study, Dolma et al. investigated how hyperoxia and a complete lack of microbiome in germ-free mice influenced pulmonary alveolar and vascular development in comparison to control non-germ-free mice. They studied alveolar morphometry, pulmonary mechanics, echocardiograms, inflammatory markers, and measures of pulmonary hypertension. Germ-free and control mice in normoxia showed no difference in these parameters. In hyperoxia, however, germ-free mice had protected lung structure and mechanics and decreased markers of inflammation compared with controls (52).

Hyperoxia evokes direct effects on T lymphocyte function but also influences the composition of the microbiome, conferring further effects on the pathogenesis of BPD. While providing higher oxygen levels is often a necessary intervention to ensure survival, understanding these effects may open up novel therapeutic avenues.

### Corticosteroids

Corticosteroids are probably the most common effective immunomodulatory therapy for BDP routinely used in clinical practice. They exert anti-inflammatory and immunosuppressive properties by inducing the activity of anti-inflammatory mediators and by directly suppressing T cell proliferation and function, respectively.

Parimi et al. analysed the effect of dexamethasone treatment on peripheral blood lymphocyte counts and subsets in preterm infants with BPD before, on days 3 and 10 and 2 weeks after treatment using flow cytometry. The absolute number and percentage of lymphocytes, T cells, and CD4 cells, as well as the CD4/CD8 ratio were significantly reduced by dexamethasone by day 3 of treatment. A reduction in CD4 cells and in the CD4/CD8 ratio persisted 2 weeks after therapy was discontinued. The absolute number of B cells increased transiently, while CD8 cells were unaffected by dexamethasone (53).

To better understand the long-term immune and endocrine effects of neonatal corticosteroid treatment for BPD, Karemaker et al. performed a retrospective matched cohort study among prematurely born children who were treated either with dexamethasone or hydrocortisone or received no corticosteroids in the neonatal period. Following *in vitro* stimulation, the ratio of T cell IFN-g/IL-4 secretion was significantly higher in the dexamethasone group than in the hydrocortisone or control groups between 8 and 9 years of age. The production of IFN-g, IL-4 and IL-10 did not differ between the hydrocortisone and the control groups. The adrenocorticotropic hormone response to an age-adapted stress test was blunted in the dexamethasone group. The results demonstrate that neonatal treatment of prematurely born children with dexamethasone but not with hydrocortisone resulted in long-lasting effects on the Th1/Th2 cytokine balance and on the hypothalamus-pituitary-adrenal axis (54).

Although corticosteroids have long been used in the clinical setting for the treatment of BPD, their effect on T lymphocyte function and multi-omic interactions in this condition merits further investigation.

### Caffeine

The CAP trial demonstrated that caffeine reduces the incidence of BPD (55). However, the underlying mechanisms are poorly understood. Originally, the beneficial effect was presumed to be related to a reduction of ventilatory days secondary to increased respiratory drive and promoting diuresis and thus facilitating extubation, but emerging evidence also suggests an immunomodulatory effect. Deregulated TGF-b signalling underlies arrested postnatal lung maturation in BPD (56). Rath et al. examined the impact of caffeine on TGF-b signalling in primary mouse lung fibroblasts and alveolar epithelial type II cells *in vitro*. They also assessed the effect of caffeine administration in the first 14 days of life *in vivo* in a hyperoxia-triggered neonatal mouse model of BPD. While caffeine reduced the expression of type I and type III TGF-b receptors and increased TGF-b signalling *in vitro*, caffeine administration neither improved nor worsened lung structure in hyperoxia-exposed mice (57). On the contrary, another group showed that in a similar mouse model following 4 days of caffeine administration, caffeine modulated angiogenic gene expression and ameliorated the pulmonary microvasculature and alveolarisation (58).

Further studies are needed to describe the beneficial impact of caffeine administration in the context of BPD pathophysiology, including its effect on the inflammatory response and tissue remodelling.

### Nutrition and Breastfeeding

Information on the influence of nutrition, and in particular, breastmilk on T lymphocytes and multi-omic interactions in BPD is extremely limited. Studies to date have mostly focused on the nutritional impact of breastfeeding in BPD. Neonates with BPD have higher nutritional (protein, calcium, phosphorus and vitamin D) and energy requirements compared to preterm babies without BPD due to 15–25% increased energy expenditure related to work of breathing. Importantly, corticosteroid treatment may alter protein metabolism and the composition of weight gain and reduce bone mineralisation (59). Infants born small for gestational age and those with postnatal growth failure are more likely to develop BPD (60). However, specific components of parenteral nutrition, for example intravenous lipid emulsions, can also increase the risk of BPD, possibly through its influence on inflammatory pathways. A recent case-control study in infants born at <32 weeks of gestation or with a birth weight of <1,500 g demonstrated that infants who developed hypertriglyceridemia following parenteral nutrition had a two-fold increased incidence of BPD and longer duration of mechanical ventilation compared to controls (61). Accordingly, the authors suggest temporary suspensions or reduction of lipid intake in case hypertriglyceridemia develops.

Our group has recently demonstrated that breastfeeding significantly impacts T cell development in the first 3 weeks of life and promotes the expansion of regulatory T cells in the circulation of breastfed term neonates compared to formula-fed babies. Furthermore, breastfed neonates showed a specific and Treg-dependent reduction in proliferative T cell responses to non-inherited maternal antigens (62). Tregs may play an important role in reducing the inflammatory response associated with BPD, and neonates who developed BPD were shown to have a lower prevalence of peripheral Tregs compared to non-BPD neonates (9). Therefore, besides its nutritional benefits, breastfeeding may have a positive impact on the incidence of BPD exploiting this and possibly other, yet undiscovered mechanisms. Indeed, Xu et al. have recently reported that a daily threshold amount of 50 ml/kg/day or more of human milk in the first 4 weeks of life was associated with lower incidence of BPD (63).

### Microaspirations

Farhath et al. demonstrated increased pepsin concentrations in tracheal aspirates of intubated preterm infants who later developed BPD in comparison to those who did not. Recovery of pepsin in tracheal aspirates was secondary to gastric aspiration, not to hematogenous spread or local synthesis in the lungs (64). The authors suggested that chronic aspiration of gastric contents may contribute to the pathogenesis of BPD, however, the mechanism remains unclear. Theoretically, the translocation of components of the gut microbiome with aspiration (the gut—lung axis) and the acidity of gastric contents provoking pneumonitis may both play a role in T cell activation and dysfunction, and eventually in the pathogenesis of BPD.

## Future Directions

The following is a non-exhaustive list of future research priorities in the pathophysiology of BPD. These points all have relevance from a multi-omics perspective and address pressing questions that will expand our understanding of this disease.

- An in-depth, systems level profiling of T cell subsets and development is needed, that will characterise the dynamics and interactions of this subset with other immune and non-immune cells. Single cell methods and a multi-omics approach will be particularly relevant to achieve this goal. The results from such studies will also help identify future predictive and therapeutic targets for various aspects of the development and progression of BPD.
- There are several limitations of studying the *in vivo* interactions on the tissue level in the premature lung. Animal studies will not necessarily be able to address all of these shortcomings. For instance, our knowledge on the role and function of TRM cells in BPD is particularly limited. While the lack of live tissue sampling from patients will remain a limitation, single-cell and multi-omic methods have the potential to answer questions regarding this important T cell population.
- Further effort is needed to identify the interactions between inflammatory mediators, the lung microbiome and mechanisms governing pulmonary tissue and vessel formation in the developing lung of preterm infants. The complex influences of multi-omic interactions on alveolarisation and vessel growth in prematurity, as well as the impairment of these processes resulting in reduced effective alveolar surface area and pulmonary hypertension in BPD merit in-depth investigation.
- The importance of the gut—lung axis has only recently been recognised in BPD. The role of breastfeeding and microaspirations as possible protective and exacerbating factors, respectively, require more detailed investigation. The underlying immunological mechanisms and multi-omic interactions deserve further attention.
- Finally, as our clinical knowledge on therapeutic approaches in BPD grow, a multi-omics approach should be employed in order to discover how normal lung development and current and future therapies—such as oxygen, antibiotics, dexamethasone, hydrocortisone, sildenafil and bosentan—influence the pathogenesis of and recovery from BPD.

## Conclusions

**Figure 1:**
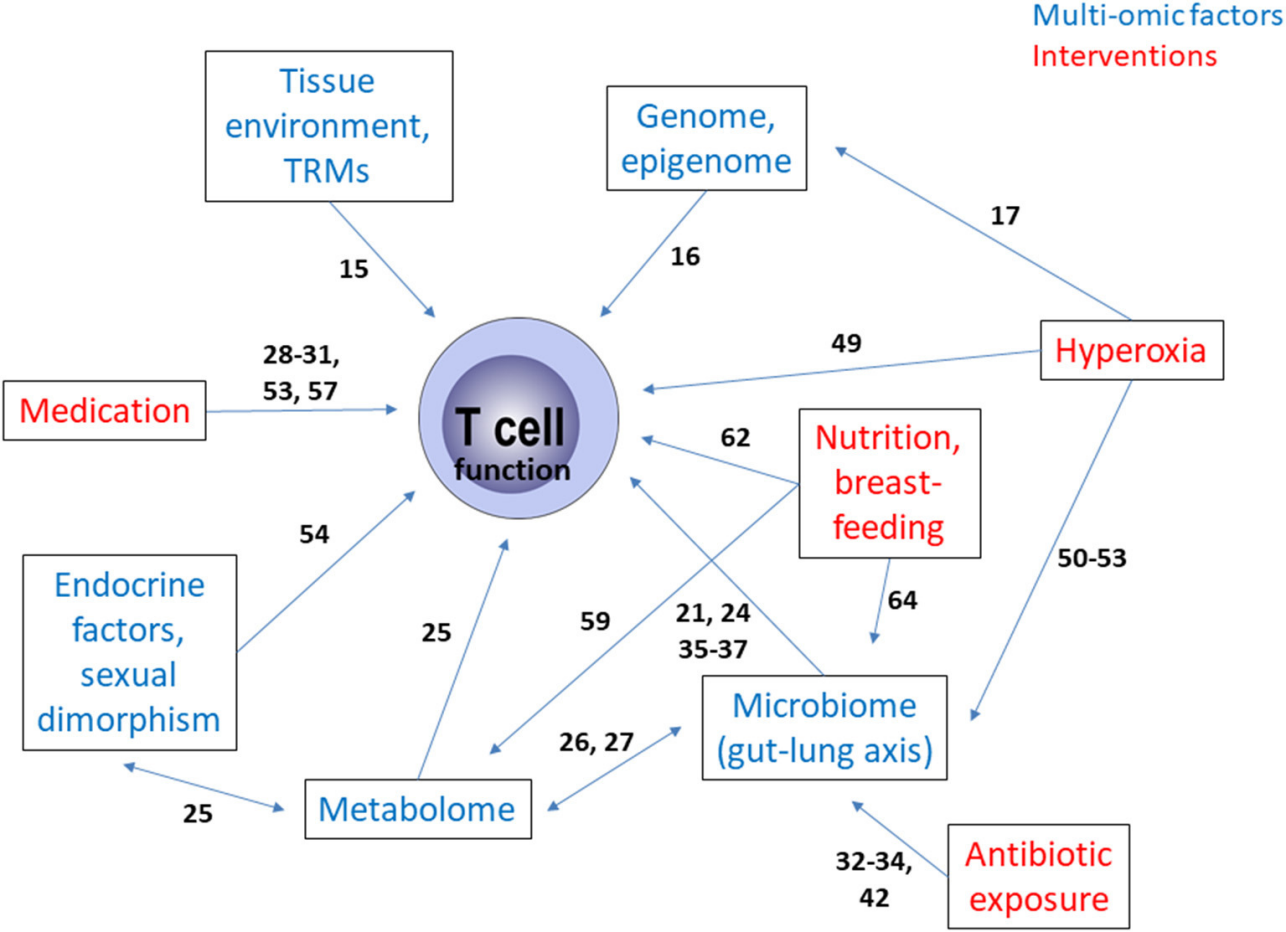
Summary of the multi-omic interactions as well as the influence of therapeutic and care interventions governing T cell function in bronchopulmonary dysplasia. The numbers on the arrows reflect the relevant literature in the reference list. TRM, tissue resident memory T cell.

In this review, our current understanding of adaptive immunity, specifically T lymphocyte development and function in relation to BPD is discussed. Interactions on several levels, with genome, epigenome, microbiome, metabolome, environmental and medical interventional factors appear to impact on this component of the immune system in BPD (Figure 1). As a disease of immaturity, with damage and repair mediated by a combination of multi-omic factors that work contiguously, it is becoming more important to understand this interplay, and the immune-mediated contribution to lung protection. A multi-omic approach is likely to be useful in future research on BPD. Immunological insights that enhance our understanding of the development of BPD, and augment the predictive value of clinical profiles of BPD in at-risk preterm neonates, may prove useful adjuncts to clinical care in the future, both for prevention and treatment of BPD.

## Author Contributions

GT and TP developed the concept for the paper. GT prepared the first draft of the manuscript. All authors contributed to discussions of content, literature search, and critical review of the manuscript.

## Conflict of Interest

The authors declare that the research was conducted in the absence of any commercial or financial relationships that could be construed as a potential conflict of interest.
